# Promoting Mental Health and Criminal Justice Collaboration Through System-Level Partnerships

**DOI:** 10.3389/fpsyt.2022.805649

**Published:** 2022-02-01

**Authors:** Don Kamin, Robert L. Weisman, J. Steven Lamberti

**Affiliations:** ^1^Institute for Police, Mental Health & Community Collaboration, Rochester, NY, United States; ^2^Monroe County Office of Mental Health, Rochester, NY, United States; ^3^Department of Psychiatry, University of Rochester Medical Center, Rochester, NY, United States

**Keywords:** mental health, criminal justice, collaboration, collaboration and organizations, criminalization

## Abstract

Recent high-profile deaths of unarmed individuals in police custody have raised concerns about the role of police officers in responding to people who are experiencing mental health crises. Of further concern, people with serious mental illness are highly over-represented throughout the entire criminal justice system including within jail, prison and community corrections populations. It is widely accepted that promoting mental health and criminal justice collaboration is a key to addressing these concerns. Promoting effective collaboration is challenging, however, due to fundamental differences in cultures and methods that exist between mental health and criminal justice service providers. To promote effective collaboration between service providers, a conceptual framework was recently published that divides the collaborative process into separate steps and outlines respective responsibilities at each step. Yet optimal collaboration between mental health and criminal justice service providers requires the support of their respective supervisors and agency heads. This paper extends previous work at the service provider level by applying the conceptual framework to promote effective collaboration at the systems level (i.e., between agencies). Barriers to inter-agency collaboration are discussed, and strategies for facilitating collaboration at each step of the collaborative process are presented.

## Introduction

Since the murder of George Floyd at the hands of Minneapolis police officer Derek Chauvin in May 2020, calls for “defunding police,” “reimagining public safety,” and “police reform” have grown stronger in the United States. Mr. Floyd's death garnered international attention, not only because of the video recording that clearly demonstrated excessive force used by police, but also because he was the latest example in a long list of Black men who died because of what many have called law enforcement's “warrior mentality” (1) combined with the longstanding inequities of policing (2). As a result, increased emphasis has been placed on examining the role of police.

As law enforcement has become the subject of increased scrutiny, one area that many communities in the United States are questioning is whether police should be the first or lone responders to individuals experiencing behavioral health crises. A primary concern relates to what can occur when law enforcement officers interact with individuals with mental illness. In addition to multiple anecdotal reports of adverse outcomes between individuals with mental illness and police (3), recent research from the US demonstrates that persons with serious mental illness are at an elevated risk of experiencing use of force and injury in their interactions with police compared to the general public (4). The facts that nearly 25% of fatal police shootings in the US involve individuals with mental illness (5) and that individuals with untreated serious mental illness are 16 times more likely to be killed by police than those without mental illness (6) further support the public's interest in alternatives to police as first responders. Recent data from the United Kingdom are consistent with US findings and highlight the disproportionate number of deaths in police custody of individuals with mental illness (7).

Beyond the physical dangers inherent in interactions between police officers and individuals in emotional crisis, such encounters also contribute to the disproportionate rate of incarceration of individuals with mental illness in the US (8), Canada (9), Australia (10), and the United Kingdom (11). Furthermore, research in the US has found that people with serious mental illness are currently over-represented within all areas of corrections including prisons, jails, probation and parole (12–14). As a result of such concerns, many in the US have called for shifting the responsibility of responding to mental health crises away from the police toward mental health service providers (15, 16).

There are myriad approaches to either replacing police as first responders to distressed persons or to providing additional support to law enforcement officers during that first response (17). One example that provides added support to police is the Crisis Intervention Team (CIT) program model (18). One aspect of CIT programs is an intensive, week-long training for police officers on recognizing and responding to mental illness and related disorders. Most prevalent in the United States where it was developed, CIT programs can also be found in Australia, Canada, and the UK. There have also been recent efforts to develop CIT programs in Liberia, West Africa (19). Co-responder models, where mental health professionals accompany police, are another example of providing support to law enforcement officers. Co-responder models can be found in the US, Australia, Canada and the UK (20). Regardless of which approach a given community pursues, successful implementation can require collaboration between individuals and agencies that have not previously collaborated in any meaningful or ongoing way.

Collaboration between criminal justice and mental health agencies is widely regarded as essential for effective management of justice-involved individuals with serious mental illness in many countries including the US (21–24), UK (25), Norway (26), and New Zealand (27). Custodial settings may provide ready opportunities for collaboration based on availability of clients, clinicians and correctional staff on-site. However, as clients approach their release dates, it is important for mental health service providers to collaborate with release planners and community corrections officers to ensure continuity of care (24).

Promoting such system-level collaboration, however, can be particularly challenging because mental health and criminal justice service providers have different values, methods and goals. For example, criminal justice professionals typically focus on fighting crime and protecting public safety while healthcare professionals generally focus on fighting disease and promoting patient health. These differences can potentially undermine the implementation and effectiveness of collaborative intervention strategies, both at the level of service providers and between the agencies they represent.

## Promoting Mental Health and Criminal Justice Collaboration

In light of the substantial differences between mental health and criminal justice professionals, Lamberti (28) proposed a conceptual framework as a guideline to promote effective collaboration at the service provider level (Table 1). While Lamberti's initial conceptualization was based on work in the US, nevertheless, we believe the basic tenets outlined have relevance to other countries. This six-step framework recognizes that although mental health and criminal justice professionals serve very different functions, the process by which they serve their respective clients has important similarities that can provide a foundation for collaboration. Specifically, they both must engage and assess their clients, they must develop and initiate service plans, they must monitor progress, they must solve problems, and they must transition their clients when a change in service intensity is indicated. As shown in Table 1, collaboration at each step can potentially improve intervention efficiency and effectiveness in serving clients with serious mental illness who are involved with the criminal justice system. Effective collaboration also requires service providers to embrace patient health and public safety as complementary rather than competing goals, and to emphasize use of problem solving over enforcement-oriented approaches. In the absence of these important philosophical underpinnings, research suggests that attempts at working together can result in increased rates of arrest and incarceration for justice-involved clients (29, 30).

**Table 1:** A collaborative framework for serving justice-involved adults with serious mental illness.

**Mental Health Service Provider Activities**	**Criminal Justice Service Provider Activities**	**Potential Benefits of Collaboration**
**Engagement**		
Discuss available treatments and services with client	Discuss legal stipulations and conditions with client	Legal leverage can promote engagement of clients who are otherwise unwilling or unable to accept necessary treatment
**Assessment**		
Conduct psychosocial assessment	Conduct criminogenic risk/need assessment	Sharing assessment results can promote a more complete understanding of client problems and potential solutions
**Planning and Treatment**		
Plan treatments and services Provide treatment	Plan supervision method and frequency Provide supervision	Coordinating planning and intervention efforts can promote intervention efficiency and effectiveness
**Progress monitoring**		
Monitor adherence to treatments and services Submit progress reports to criminal justice partner	Monitor adherence to legal stipulations and conditions Review progress reports with mental health partner	Monitoring client progress together can lay the groundwork for shared problem solving
**Problem solving**		
Consider therapeutic options Present recommendations to criminal justice partner	Consider rewards and graduated sanctions Discuss alternatives to punishment with mental health partner	Shared problem solving can promote identification of potential solutions including therapeutic alternatives to punishment
**Transition**		
Discuss transitional supports with client	Discuss termination of supervision with client	Collaborating around termination of services can promote continuity of care

The original aim of this conceptual framework has been to promote effective collaboration between service providers in managing mutual clients who straddle both the mental health and criminal justice systems. However, *optimal collaboration between service providers requires the support of supervisors and senior officials within their respective agencies*. Using the conceptual framework as a guide, we now shift the focus from service providers to collaboration between their respective agencies (i.e., system-level collaboration) to benefit justice-involved clients and those at risk for such involvement.

## Engagement

In presenting his conceptual framework, Lamberti suggested that collaborating mental health and criminal justice staff should begin by engaging their mutual clients around the common goal of being healthy and free from criminal justice involvement (28). Likewise, we propose that engagement at a systems level occurs when agencies share common goals. For example, shared goals can include the desire to have less criminal justice involvement among individuals with mental illness, improved overall health for community members, or improved public safety. Engaging different mental health and criminal justice agencies requires clarifying the respective benefits for each group. For instance, law enforcement officials are likely to express interest in initiatives that can potentially minimize the times that police are called upon as first responders for someone in an emotionally distressed state. Likewise, jail administrators are usually willing to participate in initiatives aimed at reducing incarceration of individuals with mental illness in order to avoid costs associated with psychotropic medications and 1:1 safety observations. Mental health officials, in turn, are generally interested in initiatives with the potential to reduce clients' criminal justice involvement, to reduce harmful outcomes associated with such involvement, and to improve their quality of life.

One indication of the amount of inter-agency or system-level collaboration occurring in a locality is the presence of regular meetings that are not individual or case-specific, but instead address ongoing interface issues. These meetings are typically attended by agency directors (and/or their designees) from different systems and disciplines, and they concentrate on identifying and addressing problems that prevent individuals' engagement in optimal levels of treatment. A good example of such meetings are the steering committees that are part of most Crisis Intervention Team (CIT) programs. As detailed by Usher et al. (18), CIT steering committees generally include representation from law enforcement and other criminal justice agencies, mental health providers and oversight agencies, and mental health advocacy organizations including individuals living with mental illness. The steering committee provides the infrastructure to support CIT program implementation with a goal of finding ways to transform the local crisis response system to minimize the times that police officers are called as first responders for individuals in emotional distress.

Another indication of system-level engagement is the presence of shared work products such as a Memorandum of Understanding (MOU) or other agreements that outline actions that various parties have agreed to take to address a specific issue or problem. For example, senior leaders in Monroe County, New York (USA) identified a challenge pertaining to incarcerated individuals who required inpatient psychiatric care. To address long wait times that prevented timely access to inpatient beds in state-run forensic psychiatric units where incarcerated individuals were typically referred, a protocol was developed to allow individuals to be released from custody to community-based hospitals for inpatient care. This procedure took several months to develop and required “buy-in” from multiple parties including the District Attorney's and Public Defender's Offices, the Sheriff's Department/Jail, the County Office of Mental Health, the Supervising Judge of the regional judicial district, and the Pre-Trial Services Corporation responsible for monitoring the release of such individuals. Other interested parties, including representatives from the local National Alliance on Mental Illness (NAMI) affiliate, were also part of the protocol development process.

### Facilitating System-Level Engagement

Collaboration in pursuit of shared goals at an agency level requires engagement of senior stakeholders. Therefore, the first task for facilitating system-level engagement is to identify and bring together senior representatives of local mental health and criminal justice agencies. The joining together of these and other key community stakeholders lays the groundwork for clearly articulating a shared problem or a common goal for all agencies. It is important to delineate the problem or goal in a specific enough manner to elicit interest among key stakeholders. Such delineation can benefit from review of pertinent data as further discussed in the Assessment section below.

Several national initiatives in the US provide resources that communities can use to facilitate system-level engagement. In an example of national cross-system collaboration, the National Association of Counties, the Council of State Governments Justice Center, and the American Psychiatric Association Foundation partnered to develop the “Stepping Up Initiative” to encourage local cross-system collaboration to reduce the number of people with mental illnesses in jail (31). This initiative provides step-by-step suggestions on how local communities can address the disproportionate rate of incarceration of individuals with mental illness, including a template for a “Stepping Up Resolution” that counties are required to adopt to be officially recognized as part of the national project.

Another resource for facilitating local cross-system collaboration is the Group for Advancement of Psychiatry's recent publication entitled “Roadmap to the Ideal Crisis System” (32). The authors note that all stakeholders should be engaged in crisis system design including legislators, payers, state and local policy makers, service providers, researchers, service recipients and judges. The publication includes a “Community Behavioral Health Crisis System Report Card” designed to assist communities working on enhancing their crisis system to assess their status and help prioritize next steps. In addition to these examples, the Council of State Government's *Justice & Mental Health Collaboration Program* (33) and the Bureau of Justice Assistance's *Police-Mental Health Collaboration Toolkit* (34) provide additional resources to support promotion of cross-system collaboration at the agency and community level within the US.

## Assessment

The next step of the original collaborative framework is assessment, which involves mental health professionals conducting psychosocial assessment and criminal justice professionals conducting criminogenic risk and need assessment. According to the framework, the sharing of assessment results by respective service providers can enable them to have a more complete understanding of the challenges faced by their mutual clients. From a system-level perspective, assessment refers to evaluating and defining systemic challenges within a region rather than individual challenges faced by specific service recipients. Examples of common systemic challenges include jail overcrowding, lack of access to mental health services, and lack of coordination between jail, emergency room and hospital service providers.

Challenges faced by different communities are likely to vary depending on demographic, cultural and social factors in addition to availability of local resources. Assessment of each community's unique systemic challenges requires examination of local data, ideally a combination of numerical data along with poignant anecdotes based on client experiences and first-hand accounts. In the MOU example above, data consisted of lengths of stay of incarcerated individuals awaiting placement in state-run forensic facilities, in addition to descriptions of the concerning clinical condition of these individuals as they awaited treatment. Based on these data, all stakeholders quickly saw the need for a remedy and worked collaboratively to develop a protocol to address the problem.

Having both access to and capability of analyzing local data are integral parts of assessment. In the absence of local data, however, communities can still begin the assessment process by examining national data and trends. For example, and as discussed previously, the disproportionate rate of incarceration of individuals with mental illness is a widespread phenomenon (8–11). Whatever data agency representatives ultimately decide to utilize, the processes of system-level engagement and assessment can both be facilitated through the process of Sequential Intercept Mapping.

### Facilitating Assessment of System-Level Issues

Based upon the Sequential Intercept Model (SIM) (35, 36), Sequential Intercept Mapping is a commonly used method to assess and identify challenges at the interface of the mental health and criminal justice systems. To date, the Sequential Intercept Model has been primarily used in US communities, although there is one report from Northern Ireland that incorporated the SIM structure in a literature review to address the needs of justice-involved individuals with complex needs (37). Mapping is conducted via a workshop that brings together key stakeholders with facilitators that help the group detect strengths and gaps in how local mental health and criminal justice agencies respond to people with mental illness, particularly those in crisis. The SIM mapping process takes advantage of all local data sources, both numerical and anecdotal. Identification of gaps or problems via SIM mapping helps to focus community agencies on addressing the identified issues. In addition, there is preliminary evidence that the mapping process itself increases cross-system collaboration (38).

In the absence of a formal SIM process, agency representatives can still draw upon available data including anecdotal reports from within their respective agencies. For instance, law enforcement representatives may be aware of gaps in the mental health system including the fact that police often have little or no access to mental health resources after-hours and on weekends (39). Likewise, mental health representatives may be aware of local issues pertaining to the criminal justice system, such as problematic encounters of patients with police, challenges to ongoing communication with community correctional staff or barriers to medication administration in jail settings. Such informal sources of information can provide important clues about a community's best opportunities for improvement, thus laying a foundation for intervention.

## Planning and Treatment

In the original collaborative framework, planning and treatment represent a third step in the process of collaboration between criminal justice and mental health service providers. In that context, it is important for collaborating mental health and criminal justice professionals to both use evidence-based practices to address their shared clients' mental health problems and criminogenic needs, respectively. Discussion between both professionals is also needed to decide who will be responsible for providing which treatments and services for each client. In our system-level approach, “planning and treatment” are represented by collaborative intervention strategies designed to address systemic challenges that were identified via the preceding assessment phase. Such strategies can include developing and initiating regulatory or policy changes, funding initiatives or special projects. For example, after observing an increase in people with serious mental illness within the Monroe County, New York (USA) jail, county officials initiated a grant application process to encourage mental health and criminal justice agencies to partner in addressing the problem. The result, Project Link, consisted of a consortium of mental health, correctional and social service agencies that met regularly to oversee jail in-reach activities and community-based diversion efforts with the goal of preventing unnecessary incarceration of individuals with psychotic disorders (40).

### Facilitating System Planning and Treatment

The results from SIM mapping or similar assessment processes form the foundation for system-level planning and treatment. At a service delivery level, this process involves developing and implementing individualized, person-centered “treatment plans.” At the systems level, however, the planning and intervention process typically involves formation of inter-agency workgroups as noted in the above example. In this context, the systemic “treatment” is the specific action initiated by the workgroup of senior mental health and criminal justice stakeholders. There is wide variability in the functioning of workgroups that form subsequent to identifying systemic issues or problems. Some workgroups develop specific workplans with timelines and associated milestones. Others agree to meet on an ongoing basis to address issues as they arise. Regardless of the specific workstyle, common themes across community workgroups are their cross-system membership and their aim of achieving system transformation through collaboration.

The existence of an infrastructure that enables systemic change (or “system reform” in current parlance) is an important factor that facilitates mental health and criminal justice systems collaboration. The CIT steering committees mentioned previously are one example of that infrastructure. Another example is seen in Monroe County, New York (USA), where a monthly Mental Health Criminal Justice Committee provided the necessary foundation for development of the protocol previously noted to improve access of incarcerated persons to inpatient psychiatric care. Having cross-systems stakeholders engaged in a regularly scheduled meeting provides an ongoing opportunity to address system issues as they are identified. In addition, having such a forum ensures that issues that might not rise to the level of calling a separate meeting will still be discussed, enabling a more continuous quality improvement process.

## Progress Monitoring

At the service delivery level, collaborating service providers must monitor for signs of client progress as well as non-adherence to treatment plans. Communication is a key to effective monitoring, and it ideally includes face-to-face meetings between representatives of the outpatient mental health team and supervising criminal justice agency. In contrast to focusing on individual client progress, however, progress monitoring at an inter-agency level means focusing on progress toward systemic change.

A common challenge for cross-systems committees and their workgroups is that they may have difficulty following through once the initial enthusiasm generated by joining together wanes. Progress monitoring is therefore essential both to drive the intervention process as well as to determine whether desired intervention outcomes are being achieved. This process requires monitoring of workgroup progress, a task generally accomplished by having workgroups report back to the larger cross-systems committee.

### Facilitating System Progress Monitoring

Having access to outcome data is essential for monitoring both the implementation and the effectiveness of committee-based intervention strategies. It may be helpful for cross-systems committees to adopt formal quality improvement methods (e.g., Plan-Do-Study-Act) as discussed by Rudes et al. (41). In addition, to ensure an active approach to systems change, monitoring can include review of meeting minutes to ensure that each workgroup sub-committee has clear goals, timelines and responsible parties. Once workgroups are fully engaged, it then becomes essential to have access to whatever data is necessary to help determine the effectiveness of cross-systems intervention. Depending on individual community needs and priorities, such data can include information about hospitalization or incarceration rates, frequency of adverse events, and/or data pertaining to mental health or criminal justice service costs.

A primary challenge in gathering data for progress monitoring is that mental health and criminal justice data often exist in separate repositories governed by separate administrative structures. If needed, efforts should be made to combine data sets for progress monitoring purposes. For example, cross-referencing mental health and jail databases can enable cross-system committees to track whether the proportion of psychiatric patients who become incarcerated is increasing or decreasing. Linking mental health, jail and financial databases can likewise enable cross-system committees to determine whether service costs are increasing or decreasing. In addition to enabling outcome assessment, ongoing monitoring of combined databases can promote enhanced recognition of trends, identification of emerging service gaps, and greater understanding of service recipients' needs.

Despite the potential benefits of having access to cross-system data for monitoring purposes, such access is typically lacking among collaborating agencies and cross-system oversight committees. To assist with procuring, managing and sharing cross-systems data, a detailed checklist developed by the Justice Center of The Council of State Governments in the US, can facilitate and guide the creation of a cross-system data warehouse (42). The process is divided into a three-phased approach with Planning, Development, and Implementation/Maintenance steps. According to the authors, governing groups should follow the checklist at each phase to assess their progress and then gain consensus prior to moving onto the next phase. It is further recommended that the data warehouse checklist be completed by agency leaders and other key stakeholders along with information technology (IT) staff from their respective agencies. To assist collaborating agencies in identifying progress at each phase of data warehouse Planning, Development and Implementation, rating is suggested as to whether their planned practices and policies have been developed, are underway, are planned for, or are not yet either planned for or in place.

## Problem Solving

At the service delivery level, even clients who have made good progress can still be expected to have occasional backward steps on their journey to recovery. Likewise, even communities that have developed productive, ongoing cross-system collaborations should anticipate some difficulties from time to time. In fact, problems can arise at any step of the collaborative process including engagement, assessment, planning and treatment, and progress monitoring. Understanding and addressing such problems is critical to the success of system-level collaborations.

Although communities may initially be successful at engaging key stakeholders, stakeholder engagement and participation can decline over time. For example, a committee might suddenly stop meeting due to retirement, relocation, or medical leave of the chairperson or another individual who served as the committee's main organizing force. Alternately, attendance and participation of committee members may gradually dwindle over time if a committee loses its focus. Loss of focus can occur if a committee has failed to conduct an adequate assessment of challenges to be addressed, leading to inadequate understanding of the problem and failure of planned interventions. Likewise, committee members can become disengaged in the absence of progress monitoring data to track effectiveness of their planned interventions in an ongoing way.

### Facilitating System-Level Problem Solving

Understanding the reasons behind a committee's lack of progress can serve as an important first step in facilitating problem resolution. In some instances, the primary cause is obvious and thus a remedy is usually easily identifiable. If a committee stops meeting because of the chairperson's departure, for example, then it is incumbent that someone step forward to call a meeting to discuss identifying a new chairperson or possibly co-chairs. Diffusion of responsibility (43) is an important barrier to recognize in these situations; committee members may be less apt to take action because each individual defers to others in the group. In addition, some may feel that taking the initiative to organize a meeting could inadvertently lead them to becoming burdened with the role of chairperson. One potential solution is for the agency affiliated with the former chairperson to assume responsibility for filling that role. Alternatively, cross-system committees can utilize a yearly rotation whereby the role of chairperson rotates between different criminal justice and behavioral health agencies, thus ensuring a line of leadership succession. Other common internal issues that can present barriers to successful system interventions involve committee meeting frequency and meeting duration. In such instances committee leadership must ensure that meetings are neither so frequent or lengthy as to be burdensome nor so infrequent or brief as to undercut a committee's momentum.

Changes outside of the control of committee workgroups can also create obstacles to progress. An example is the current global COVID-19 pandemic. Even as most community agencies became accustomed to the advantages of employing virtual platforms for meetings, the focus of many cross-system workgroups shifted toward addressing emergent issues related to COVID-19 management. While external events may distract committee workgroups from their agendas, the experience of working together to address such challenges can strengthen collaborative bonds and provide a foundation for addressing future priorities.

At other times it is less clear why the goals of a cross-system committee are not being met. In those instances it may be helpful to re-evaluate the purpose, composition, and structure of the committee. Some system-level committees are initiated for a specific purpose and over time drift from that initial focus. Rigid adherence to initial priorities is not necessary, however, as long as interventions have been planned and implemented to address the original goals of the group. Once a cross-system committee has achieved originally intended goals, then the committee can be understood as entering the transition stage of collaboration.

## Transition

Transition is the final step of the collaborative framework. At the level of mental health and criminal justice service providers, this phase involves transitioning clients to less intensive mental health treatment and/or less intensive criminal justice supervision depending on clients' current involvements. At the systems level, the nature of the transition phase will depend on the nature of the cross-systems collaboration. If a collaboration is highly focused and time-limited as with grant-funded projects, then transition might involve securing continuation funding to ensure project sustainability. If collaboration involves standing committees or workgroups, then this phase will likely involve transitioning from one area of concern to the next in a manner consistent with continuous quality improvement.

### Facilitating System Workgroup Transitions

Facilitating such transitions likewise depends on the nature of the collaboration. In general, time-limited initiatives require collaborators to anticipate what resources will be needed to sustain their progress. Along with the possibility of needing continuation of funding, such resources can involve personnel, facilities or administrative or regulatory considerations. In comparison to time-limited collaborations, standing committees are usually less concerned with ensuring the ongoing success of a single initiative. Rather, their successful transitions from one initiative to the next can be facilitated by having clear methods for identifying and prioritizing workgroup goals.

## Discussion

Collaboration between mental health and criminal justice professionals is generally viewed as essential for serving people with serious mental illness who are involved in both service systems. Yet developing effective collaborations can be challenging given the substantial differences that exist between mental health and criminal justice service providers. To promote collaboration between service providers, a collaborative framework was published in 2016 which divided the collaborative process into six separate stages with respective mental health and criminal justice activities at each stage. This framework was applied in a randomized controlled trial of forensic assertive community treatment (FACT) that required collaboration between treatment team clinicians and a criminal court judge (44). FACT was effective at reducing hospitalizations, convictions and jail time, and the experience of conducting the study along with their experiences as FACT consultants (45) raised the authors' awareness of the importance of gaining agency-level support for optimal service-level collaboration.

Service providers are accountable to their parent agencies for following applicable policies and procedures and for pursuing their respective agency missions. In the absence of shared institutional goals and priorities, collaborating service providers may find themselves working at cross purposes to the detriment of their mutual clients. Having agency or department-level support of collaboration creates a culture and expectation that personnel from different agencies across the mental health and criminal justice systems will collaborate for the benefit of individual clients. In the absence of system-level engagement between mental health and criminal justice agencies, effective case-specific collaboration is less likely to occur at the individual client level. Building upon the 2016 collaborative framework for service provider collaboration, this paper presents a framework for promoting effective inter-agency collaboration. Research is needed to examine the effectiveness of such collaboration in promoting positive mental health and public safety outcomes in serving justice-involved adults with serious mental illness. In addition, more work is needed to determine to what extent this approach, developed from our US-based work, is applicable to other countries.

## Author Contributions

All authors made substantive contributions to the manuscript and have approved the final submitted version.

## Conflict of Interest

The authors declare that the research was conducted in the absence of any commercial or financial relationships that could be construed as a potential conflict of interest.

## Publisher's Note

All claims expressed in this article are solely those of the authors and do not necessarily represent those of their affiliated organizations, or those of the publisher, the editors and the reviewers. Any product that may be evaluated in this article, or claim that may be made by its manufacturer, is not guaranteed or endorsed by the publisher.
